# Application of ATR-FTIR Spectroscopy for Analysis of Salt Stress in Brussels Sprouts

**DOI:** 10.3390/metabo14090470

**Published:** 2024-08-26

**Authors:** Su-Min Yun, Cheol-Soo Kim, Jeung-Joo Lee, Jung-Sung Chung

**Affiliations:** 1 Division of Applied Life Science, Gyeongsang National University, Jinju 52828, Republic of Korea; 2 Department of Applied Biology, Chonnam National University, Gwangju 61186, Republic of Korea; cskim626@chonnam.ac.kr; 3 Department of Plant Medicine, Institute of Agriculture and Life Science, Gyeongsang National University, Jinju 52828, Republic of Korea; jeunglee@gnu.ac.kr; 4 Department of Agronomy, Institute of Agriculture and Life Science, Gyeongsang National University, Jinju 52828, Republic of Korea

**Keywords:** FTIR spectroscopy, metabolic changes, photosynthetic efficiency, salt stress

## Abstract

Salt stress is one of the environmental stresses that significantly reduces crop productivity and quality worldwide. Methods to overcome salt stress include developing salt-resistant crops by inserting various resistance genes or to diagnosing and responding to the effects of salt stress at an early stage. In this study, we investigate the effects of salinity stress on growth, photosynthetic efficiency, and metabolic changes in Brussels sprouts (*Brassica oleracea* var. gemmifera). Fresh weight and leaf area decreased significantly with increasing NaCl concentration, indicating that salinity stress has a detrimental effect on plant growth. However, chlorophyll fluorescence parameters did not show significant changes, suggesting that photosynthetic efficiency was not significantly affected over 10 days. Fourier transform infrared (FTIR) spectroscopy revealed notable metabolic adjustments, especially in lipids, plastids, proteins, and carbohydrates, indicating biosynthesis of protective compounds such as anthocyanins and proline in response to salinity stress. Pearson correlation analysis confirmed a strong relationship between NaCl concentration and the observed physiological and metabolic changes. The findings highlight the potential of FTIR spectroscopy as a non-destructive tool for early detection of salinity stress and timely intervention to improve crop resilience and yield. This study highlights the widespread application of FTIR spectroscopy in agricultural research to manage abiotic stresses in crops.

## 1. Introduction

Cruciferous vegetables are well-recognized for their high levels of vitamins, antioxidants, and compounds with anticancer properties [1,2,3]. Among these vegetables, Brussels sprouts (*Brassica oleracea* var. gemmifera) stand out due to their exceptionally high content of vitamins C and A, significantly exceeding the levels found in cabbage. Moreover, Brussels sprouts have been shown to contain greater amounts of antioxidant substances, such as phenols and flavonoids, compared to regular cabbage [4]. They also possess substantial quantities of glucosinolates, known for their anticancer effects [5]. This increased awareness of their health benefits has led to a rise in consumption in Korea, with imports growing from approximately 60 tons in 2014 to about 200 tons in 2022 [6,7].

In response to the rising demand, the cultivation of Brussels sprouts in Korea has expanded, particularly in Jeju and highland areas. However, these regions present unique challenges: Jeju’s proximity to seawater increases the risk of salt damage, while highland cultivation requires effective thermal insulation of seedlings during early growth stages and often necessitates facility-based cultivation. In domestic facility cultivation areas, salt damage is notably severe, indicating that the cultivation environment for Brussels sprouts is highly vulnerable to salt stress.

Salt stress is known to induce various metabolic disturbances in plants, impairing growth and photosynthesis, which subsequently leads to reduced yield [8,9]. To mitigate the effects of salt stress, plants enhance the biosynthesis of metabolites such as anthocyanins and proline [10,11]. Thus, salt stress significantly influences the photosynthetic and metabolic processes of crops. Chlorophyll fluorescence, which is measured when photosystem II in chlorophyll receives light, transfers it to photosystem I, and emits the remaining energy as fluorescence, is a widely used indicator of abiotic stress in plants [12,13,14]. These parameters, assessed after dark adaptation of leaves for about 30 min, offer a convenient and non-destructive method to evaluate photosynthetic responses in plants, even at small sizes.

Fourier Transform InfraRed (FTIR) spectroscopy, and specifically Attenuated Total Reflection (ATR-FTIR), is a robust technique for identifying bonds between elements in a sample by detecting specific infrared absorption wave numbers [15,16]. ATR-FTIR involves placing a sample on a window, irradiating it with infrared rays from below, and analyzing the reflected rays. This method is advantageous due to its requirement of only a small sample size and its short measurement time of approximately one minute [15]. FTIR is a highly sensitive analytical method that offers several advantages over other techniques, particularly due to its non-destructive nature and its ability to identify functional groups. This capability provides valuable insights into structural and chemical changes in plants that are associated with various biotic and abiotic stresses. For instance, significant alterations in the FTIR spectrum, specifically in the C-H bond region of lipids and plastids, have been observed in peas subjected to high-temperature stress [17]. Similarly, changes in the FTIR spectrum have been linked to increased chlorophyll content in plastids when maize was treated with phosphorus [18]. Additionally, salt stress applied to ice plants resulted in detectable peak shifts in the amide region, related to proteins, within the 1800–1500 cm^−1^ spectral range of FTIR [19]. Furthermore, FTIR spectroscopy has been employed to estimate the ratio of protein secondary structures in maize pollen [20]. Despite the extensive research utilizing FTIR, the changes in chemical composition due to salt stress remain insufficiently understood, and the correlation between these changes and physiological conditions in plants is not yet well established. This gap highlights the need for further investigation into the use of FTIR to elucidate the chemical and physiological responses of plants to salt stress.

This study aims to observe changes in photosynthesis and metabolites in the early stages of Brussels sprout growth under salt stress using chlorophyll fluorescence and ATR-FTIR spectroscopy. Furthermore, if metabolite changes can be detected using ATR-FTIR, this technique could potentially be utilized to identify other abiotic stresses in the future. By analyzing metabolites in small sample amounts during early growth stages, this method may enable the early identification of stress conditions.

## 2. Materials and Methods

### 2.1. Experimental Materials and Growth Environment

Brussels sprouts seeds were purchased from Asia Seed Korea. It is an early maturing variety with strong cold tolerance, but its salt tolerance has not yet been reported. Brussels sprout seeds were initially sown in 128 plug trays and allowed to grow for 30 days. Subsequently, they were transplanted into circular pots (9 cm × 8 cm) and cultivated for an additional 15 days, using Heungnong Bioplug as the topsoil. The Brussels sprouts, after a total of 45 days of growth, were subjected to a salt treatment for 15 days using NaCl. All cultivation processes were conducted in a greenhouse at Gajwa campus farm in Gyeongsang National University.

### 2.2. Experimental Design and Treatment

Salt stress was induced by treating the plants with NaCl at concentrations of 0 (control), 50, 100, 200, 300, and 400 mM. Each plant received 50 mL of the respective NaCl solution every 2 days for a duration of 14 days. The experiment was replicated three times, with three plants per salt concentration, and three plants were used per treatment for measurements and sampling. Data presented are averages of these replicates. Sampling and measurements were performed at two-day intervals, coinciding with the treatment days, utilizing the entire plant, including roots, for sampling.

### 2.3. Growth and Chlorophyll Fluorescence Analysis

To assess the growth status of Brussels sprouts under salt stress, fresh weight and total leaf area were measured. The fresh weight of the aerial parts was recorded after severing the roots. Total leaf area was determined using the ImageJ v2.14.0 software (National Institutes of Health, MD, USA) by analyzing overhead photographs of the plants [21]. Additionally, to evaluate the impact of salt stress on photosynthesis, the uppermost fully developed leaves were dark-adapted for 30 min before chlorophyll fluorescence was measured using the OJIP test with an OS30p+ fluorometer (Opti-Science, Hudson, NH, USA). Key parameters measured included F_v_/F_m_, F_v_/F_0_, ABS/RC, and TR0/RC [22].

### 2.4. Analysis of Metabolite Changes Using ATR-FTIR Spectroscopy

The sampled Brussels sprouts were separated into leaves and roots, freeze-dried, and prepared for FTIR analysis to evaluate changes in metabolites due to salt stress. Analysis was performed using a Nicolet iS50 Spectrometer (Thermo Fisher Scientific, Waltham, MA, USA) with the ATR method. Spectra were measured in the range of 400 to 4000 cm^−1^ using the OMNIC 8.0 software (Thermo Fisher Scientific, Waltham, MA, USA), with each spectrum representing the average of 32 scans at a resolution of 4 cm^−1^ [18,23,24]. The wavenumber range of elemental bonds present in this range is shown in Table 1. Spectrum analysis was conducted using OriginLab Pro 2023b software (OriginLab Corporation, Northampton, MA, USA).

### 2.5. Pearson Correlation Coefficient and Statistical Analysis

The relationship between NaCl concentration and the experimental data was analyzed using Pearson correlation coefficients with OriginLab Pro 2023b software. The significance of all data was verified through one-way ANOVA of the NaCl concentration measurements within each treatment day, followed by the Tukey test at a 5% significance level.

## 3. Results and Discussion

### 3.1. Changes in Fresh Weight and Leaf Area of Brussels Sprouts

**Figure 1:**
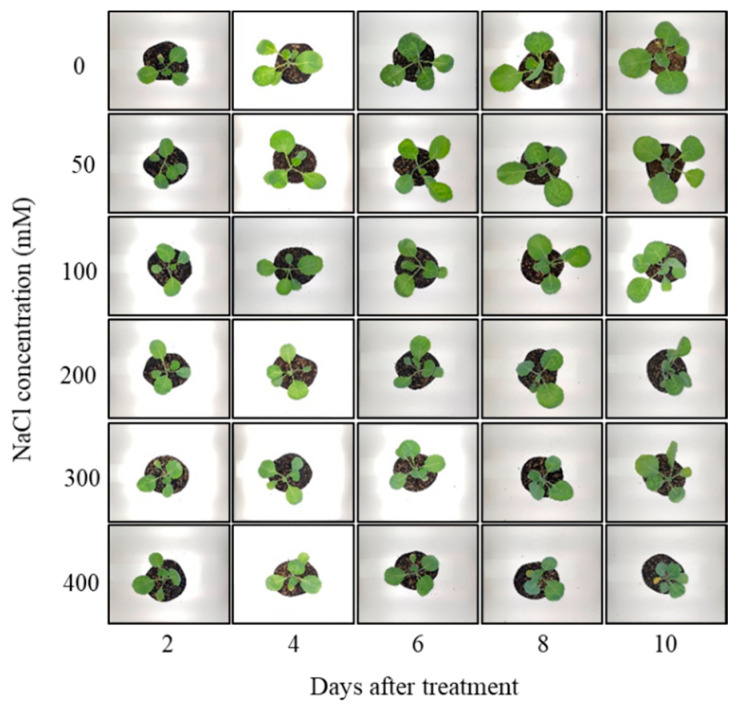
Photographs used to measure total leaf area of Brussels sprouts under different NaCl concentrations.

**Figure 2:**
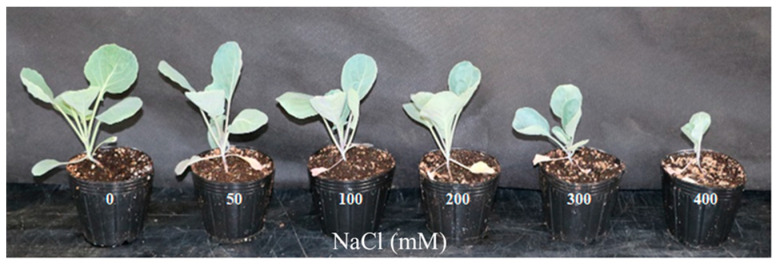
Growth of Brussels sprouts 15 days after treatment at various NaCl contents. Brussels sprouts plants cultivated in soil were treated with different NaCl concentrations.

**Figure 3:**
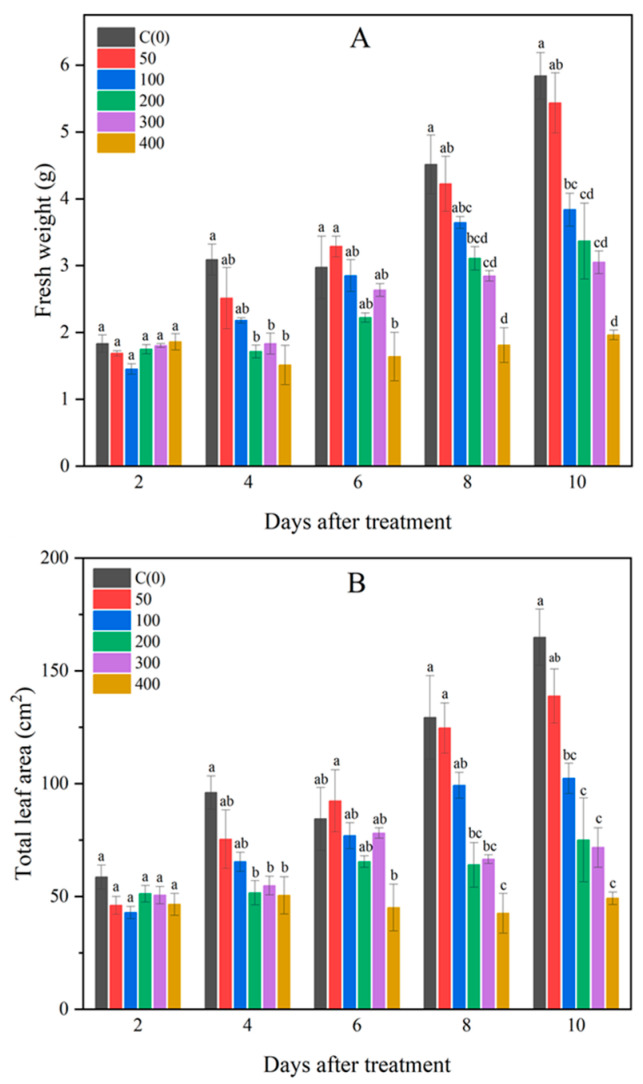
Fresh weight (**A**) and total leaf area (**B**) of Brussels sprouts under different NaCl concentrations. Different lowercase letters are significantly different among treatment at *p* ≤ 0.05 level.

Salt stress is known to cause metabolic disturbances and reduced photosynthetic rates in plants, which ultimately lead to stunted growth and decreased yield [8]. In this study, images of Brussels sprouts indicated that received increased NaCl concentrations corresponded with smaller plant sizes (Figure 1 and Figure 2). From the fourth day of NaCl treatment, a significant reduction in fresh weight was observed with increasing NaCl concentration (Figure 3A). Similarly, leaf area exhibited a substantial decrease as NaCl concentration increased from the fourth day of treatment (Figure 3B). The differences in both fresh weight and leaf area between treatments became more pronounced over time. At NaCl concentrations above 200 mM, although both fresh weight and leaf area continued to decline, the differences were not statistically significant. These findings are consistent with previous studies that reported reductions in fresh weight and leaf area due to NaCl treatment [30,31]. These results suggest that the growth of Brussels sprouts was adversely affected by NaCl treatment over the 10-day period.

### 3.2. Changes in Chlorophyll Fluorescence Parameters

Salt stress also adversely affects photosynthesis in crops [9,32]. Previous studies have shown that salt stress increases the F_0_ value and decreases the Fm value in crops [33], with the increase in F_0_ value linked to the inactivation of photosystem II [34]. Specifically, in cruciferous crops like rapeseed, salt stress has been reported to reduce F_v_/F_m_ values [35]. Based on these findings, we measured F_v_/F_m_ and F_v_/F_0_ values to assess the quantum efficiency of photosystem II in Brussels sprouts under varying NaCl concentrations. Additionally, we evaluated ABS/RC (degree of light absorption by photosystem II) and TR_0_/RC (energy capture efficiency of photosystem II) (Table 2).

The results indicated no significant differences in chlorophyll fluorescence parameters over the 10-day NaCl treatment period. Although a significant difference in quantum efficiency was observed on the eighth day, all values remained within the normal range for non-stressed plants [36,37,38]. Thus, detecting changes in Brussels sprouts under NaCl treatment using chlorophyll fluorescence parameters was challenging within this period. This observation aligns with Bacarin et al., who found no significant changes in ABS/RC and TR0/RC values in rapeseed under salt stress [39], and Shin et al., who observed no significant differences in F_v_/F_m_ values in lettuce except at a NaCl concentration of 400 mM [13]. Conversely, Kaouther et al. reported a decrease in F_v_/F_m_ in peppers under salt stress [40], suggesting that the impact of salt stress on photosystem II varies among crops. For Brussels sprouts, chlorophyll fluorescence parameters did not exhibit a sensitive response to salt stress, implying that their photosynthetic process is not severely impacted. This resilience is evidenced by the plants’ survival even at high NaCl concentrations of 400 mM over a 10-day period, indicating a degree of salt tolerance.

### 3.3. FTIR Spectrum Analysis of Brussels Sprout

**Figure 4:**
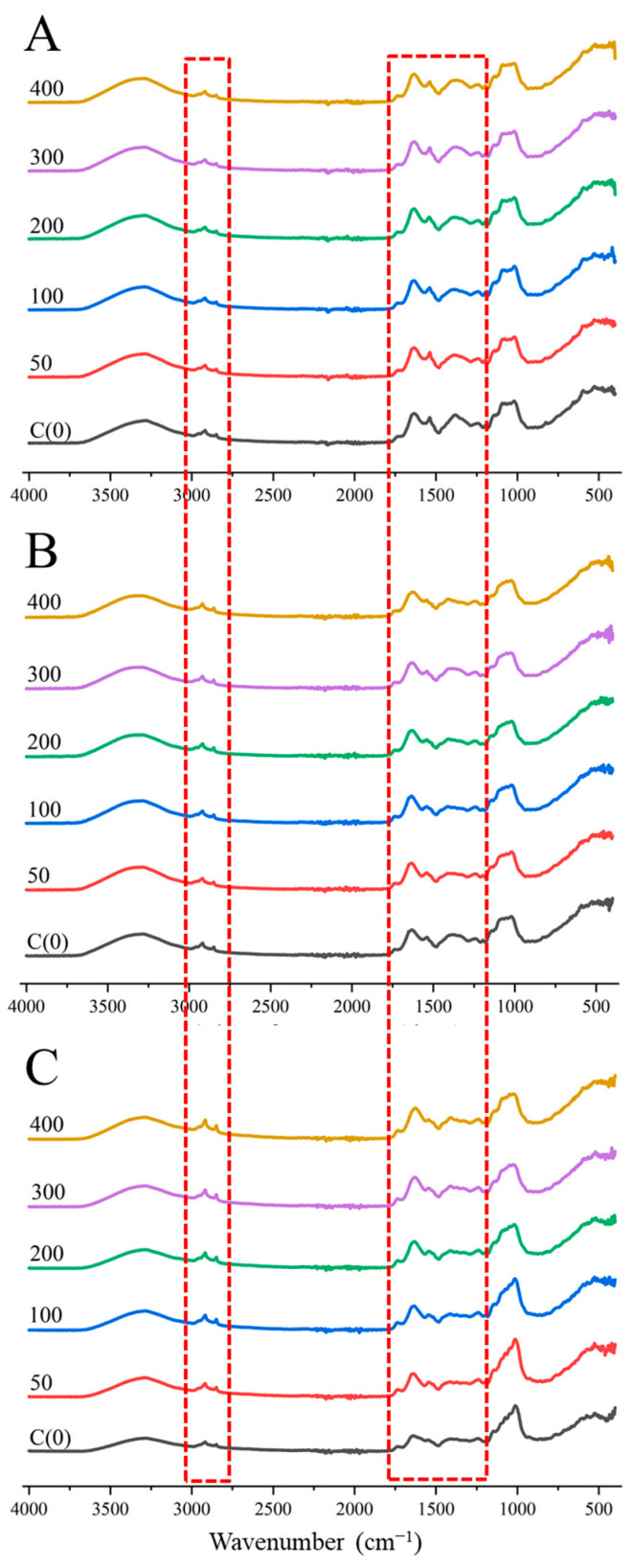
FTIR spectra of shoots in Brussels sprouts under different NaCl concentrations at the different treated periods. Y axis was offset for comparison. Spectra represent 2 days (**A**), 6 days (**B**), and 10 days (**C**) after treatment. The red dotted box indicates the part showing the difference in the spectrum.

**Figure 5:**
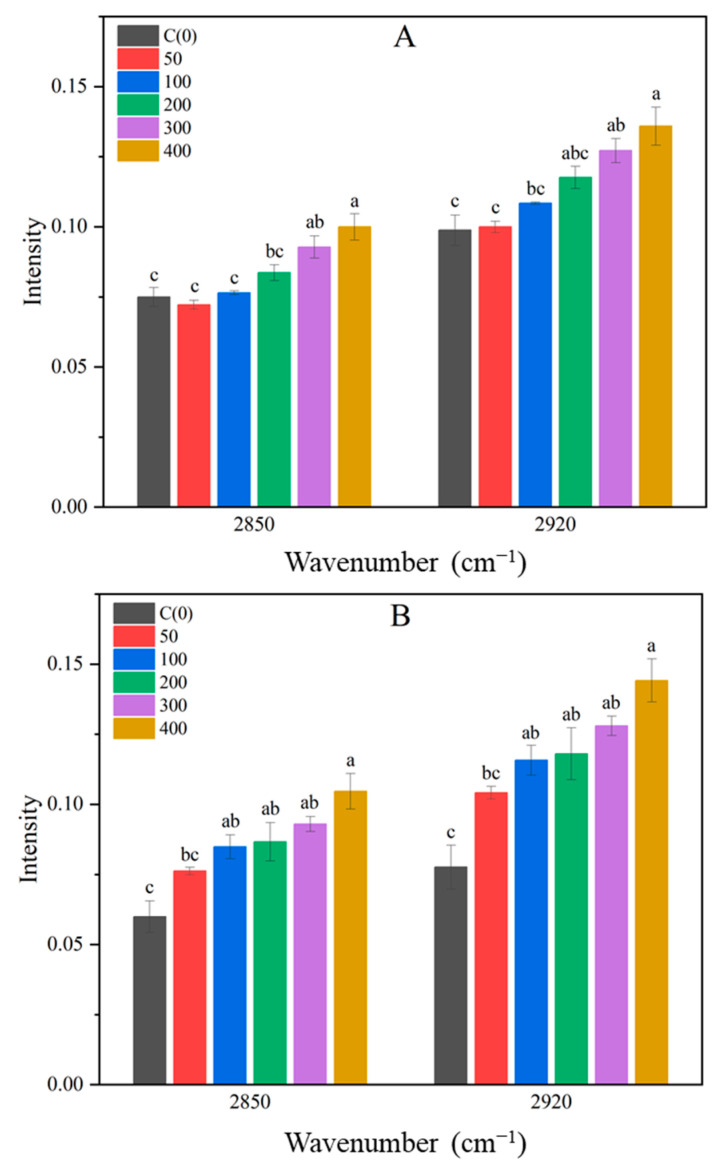
Intensity of wavenumber 2850 and 2920 cm^−1^ in shoots spectra of Brussels sprouts at 8 days after treatment (**A**) and 10 days after treatment (**B**). Different lowercase letters are significantly different among treatments at *p* ≤ 0.05 level.

Crops exposed to salt stress mitigate damage by adjusting osmotic pressure through the synthesis of organic substances and protecting themselves via various metabolic processes [9,41]. Consequently, salt stress influences the metabolic processes in crops. The leaves and roots of Brussels sprouts were analyzed to observe changes in these metabolites. Using FTIR spectroscopy, the samples were analyzed in the range of 400 to 4000 cm^−1^. The wavenumber range of elemental bonds present in this range is shown in Table 1. FTIR analysis of Brussels sprout roots showed no significant difference. Differences in the FTIR spectrum of Brussels sprout leaves began to emerge from the eighth day of treatment (Figure 4). These differences were primarily observed in two regions: the 2850 and 2920 cm^−1^ areas corresponding to lipids and plastids, and the 1200 to 1700 cm^−1^ area corresponding to proteins and carbohydrates. The peaks in the 2850 and 2920 cm^−1^ regions, related to C-H bonds of lipids and plastids, became more pronounced with increasing NaCl concentrations on both the eighth and tenth days after treatment (Figure 5). Plants generate reactive oxygen species (ROS) under salt stress due to ion toxicity and inhibited water absorption [42,43]. Chen et al. observed that the expression of the *NtCHS1* gene was promoted in tobacco treated with 200 mM NaCl, and found that deletion of this gene reduced anthocyanin and rutin levels, both of which help remove ROS [44,45]. Other studies have reported increased biosynthesis of anthocyanins under salt stress [10,46], suggesting that plants enhance anthocyanin biosynthesis when exposed to salt stress [47]. Thus, in Brussels sprouts, salt stress likely promotes the biosynthesis of plastids like anthocyanins, with higher NaCl concentrations resulting in stronger peaks at 2850 and 2920 cm^−1^.

**Figure 6:**
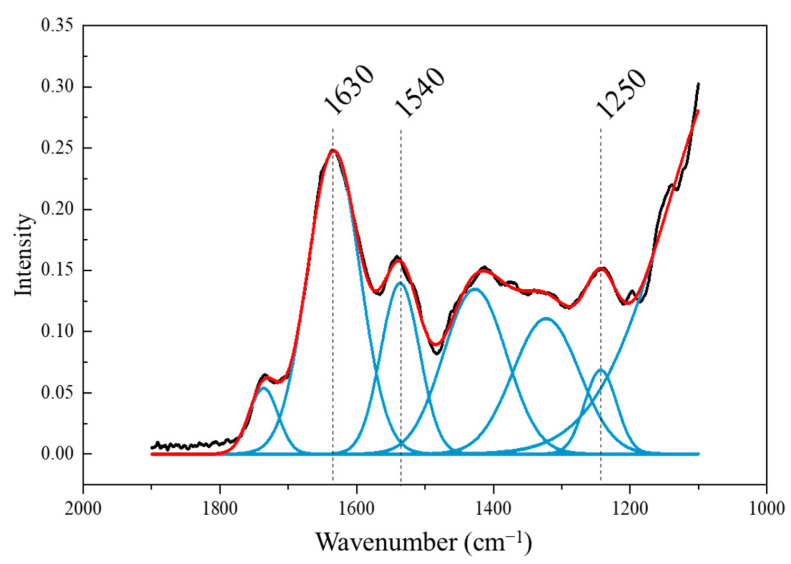
Deconvoluted peaks of Brussels sprouts shoots between wavenumber 1100 and 1900 cm^−1^ (black line, original FTIR spectrum; red line, cumulative spectrum of deconvoluted peaks; blue line, deconvoluted peak; 1630 cm^−1^, amide I; 1540 cm^−1^, amide II; 1250 cm^−1^, amide III).

**Figure 7:**
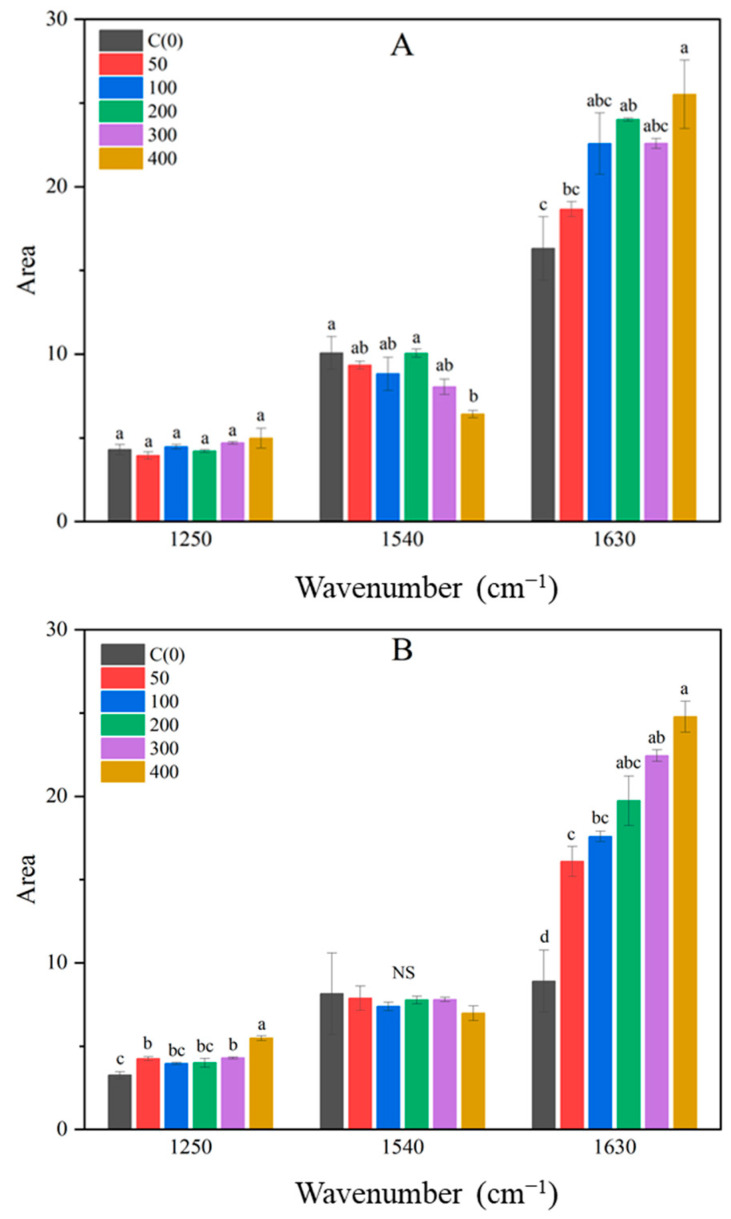
Area of amide I (1630 cm^−1^), II (1540 cm^−1^), and III (1250 cm^−1^) peaks in deconvoluted spectra of Brussels sprouts shoots at 8 days after treatment (**A**) and 10 days after treatment (**B**). Different lowercase letters are significantly different among treatments at *p* ≤ 0.05 level.

The 1200−1700 cm^−1^ region of the FTIR spectrum, where several peaks overlap, was deconvoluted to reveal six distinct peaks (Figure 6, Figures S1 and S2). Among these, the peaks at 1250, 1540, and 1630 cm^−1^ showed changes with NaCl treatment. The 1250 cm^−1^ peak corresponds to amide III (C-N bond), 1540 cm^−1^ to amide II (N-H bond), and 1630 cm^−1^ to amide I (C=O bond) [18,23,27]. The areas of these peaks showed significant differences on the eighth day after treatment at 1540 and 1630 cm^−1^, and on the tenth day at 1250 and 1630 cm^−1^ (Figure 7). Except for the 400 mM NaCl treatment at 1540 cm^−1^, no significant differences were observed. However, the peak areas at 1250 cm^−1^ increased with higher NaCl concentrations on the tenth day, and the peak areas at 1630 cm^−1^ increased on both the eighth and tenth days of treatment. Changes in amide peaks due to salt stress have also been observed in mustard and ice plants [18,48]. Javed et al. reported that salt stress in rapeseed led to overexpression of proteins related to energy metabolism and cell signaling [49]. It is known that proline biosynthesis is promoted under salt stress [11,50,51], which helps reduce ROS and protect the photosynthetic process [11]. Therefore, the increase in peak areas at 1250 and 1630 cm^−1^ in Brussels sprouts may be attributed to the enhanced synthesis of proteins related to proline, energy metabolism, and signal transduction under salt stress. As NaCl concentration increased, the content of plastids and proteins in Brussels sprouts also increased, detectable through FTIR spectra from the eighth day after treatment.

### 3.4. Comparison of Pearson Correlation Coefficients

**Figure 8:**
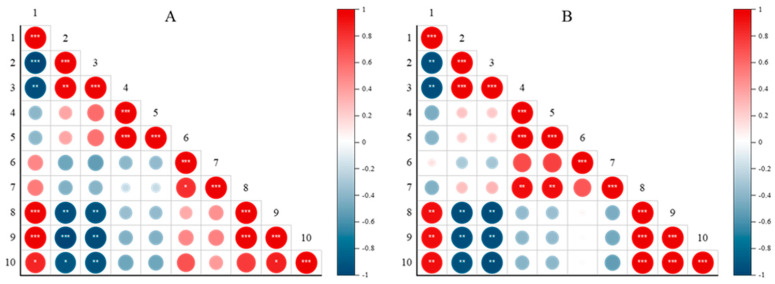
Pearson’s correlation coefficient matrices comparing treatment and measurements at 8 days after treatment (**A**) and 10 days after treatment (**B**). The degree of correlation is displayed by dot size (closer to red indicates a positive correlation, and closer to blue indicates a negative correlation). The treatment and measurements used for comparison are 1, NaCl concentration; 2, fresh weight; 3, total leaf area; 4, F_v_/F_m_; 5, F_v_/F_0_; 6, ABS/RC; 7, TR_0_/RC; 8, 2850 cm^−1^ peak intensity; 9, 2920 cm^−1^ peak intensity; 10, 1630 cm^−1^ peak area. The significance of the correlation is indicated by the label “*” inside the dots (* 0.01 < *p* ≤ 0.05, ** 0.001 < *p* ≤ 0.01, *** *p* ≤ 0.001).

To determine the relationship between NaCl concentration and the measured data, Pearson correlation coefficients were analyzed (Figure 8). On both the eighth and tenth days after treatment, increased NaCl concentration correlated with reduced Brussels sprout growth, as indicated by significant negative correlations with fresh weight and leaf area (R ≈ −0.97, R ≈ −0.95). However, chlorophyll fluorescence parameters did not show a significant correlation with NaCl concentration. In the FTIR spectra of Brussels sprout leaves, peaks at 2850 and 2920 cm^−1^ showed significant positive correlations with NaCl concentration on both the eighth and tenth days after treatment (R ≈ 0.96, R ≈ 0.97), and the peak area at 1630 cm-1 also exhibited a significant positive correlation (R ≈ 0.9). These differences became more evident over time. Thus, it can be inferred that changes in the FTIR spectrum of Brussels sprout leaves were influenced by NaCl, showing positive correlations with its concentration. Consequently, FTIR spectroscopy could potentially be used to detect salt stress in the early stages of growth, enabling timely responses to mitigate its negative effects. Furthermore, if ATR-FTIR spectroscopy can detect changes due to other abiotic stresses early on, it could help reduce damage from various stressors.

## 4. Conclusions

This study investigated the effects of salt stress on growth, photosynthetic efficiency, and metabolic changes in Brussels sprouts. Our findings revealed that increased NaCl concentrations significantly reduced both the fresh weight and leaf area of Brussels sprouts, demonstrating the adverse impact of salt stress on plant growth. Despite these reductions, chlorophyll fluorescence parameters did not show significant changes, indicating that the photosynthetic efficiency of Brussels sprouts was not severely affected by salt stress within the 10-day period. FTIR spectroscopy provided deeper insights into the metabolic adjustments in response to salt stress. Significant changes in the FTIR spectra of both roots and leaves were observed, particularly in regions corresponding to lipids, plastids, proteins, and carbohydrates. These changes suggest that salt stress prompts the biosynthesis of protective compounds such as anthocyanins and proline, aiding the plants in mitigating the effects of ROS generated under stress conditions. The correlation analysis further confirmed the relationship between NaCl concentration and the observed physiological and metabolic changes. The strong positive correlation between NaCl concentration and specific FTIR spectral peaks highlights the potential of FTIR spectroscopy as a non-destructive tool for early detection of salt stress in Brussels sprouts. This technique could facilitate timely interventions to mitigate the adverse effects of salt stress, thereby improving crop resilience and yield. Overall, our study underscores the utility of FTIR spectroscopy in detecting early metabolic changes under abiotic stress and suggests its broader application in agricultural research for stress management in crops.

## Figures and Tables

**Table 1:** The assignment of absorption bands ranging wavenumber 400~4000 cm^−1^ for FTIR spectroscopy.

Wavenumber (cm^−1^)	Band Assignments	References
900~1200	Fingerprint region of carbohydrate	[18,23,24]
1250	C-N band (amide III)	[18]
1400	Asymmetric N-H band of NH^4+^	[25]
1455	Asymmetric deformation band of CH_3_ and CH_2_ (protein)	[26]
1540	N-H band (amide II)	[18,23]
1600~1700	C=O band (amide I)	[18,23,27]
1740	C=O band of COOH (lipids and fatty acids) C-OH band of COOH	[23,28]
2850, 2920	C-H band (lipid and plastid structure)	[17,29]
around 3400	O-H and N-H band (protein and carbohydrate)	[18]

**Table 2:** Chlorophyll fluorescence parameters of Brussels sprouts under different NaCl concentrations.

Parameter	Treatment	Days after Treatment				
		2	4	6	8	10
F_v_/F_m_	C(0)	0.740	0.769	0.783	0.797 a*	0.781
	50	0.753	0.778	0.791	0.801 a	0.773
	100	0.753	0.773	0.784	0.797 a	0.782
	200	0.729	0.767	0.789	0.767 b	0.789
	300	0.749	0.786	0.787	0.781 ab	0.764
	400	0.738	0.792	0.790	0.794 ab	0.772
	*p*-value	0.5593	0.0802	0.9791	0.0095	0.3321
		2	4	6	8	10
F_v_/F_0_	C(0)	2.859	3.339	3.621	3.946 a	3.584
	50	3.105	3.525	3.803	4.027 a	3.419
	100	3.061	3.425	3.640	3.956 a	3.611
	200	2.693	3.317	3.744	3.297 b	3.752
	300	3.002	3.700	3.750	3.579 ab	3.279
	400	2.842	3.819	3.804	3.883 ab	3.408
	*p*-value	0.5170	0.0805	0.9785	0.0139	0.3229
		2	4	6	8	10
ABS/RC	C(0)	1.781	1.903	1.850	1.678	1.854
	50	2.050	1.895	1.851	1.781	1.701
	100	2.158	1.595	1.910	1.832	1.856
	200	1.779	1.722	1.875	1.802	1.988
	300	2.208	1.688	1.869	1.848	1.816
	400	1.760	1.659	1.898	1.780	1.818
	*p*-value	0.1644	0.3388	0.9963	0.9876	0.7585
		2	4	6	8	10
TR_o_/RC	C(0)	0.232	0.239	0.247	0.225	0.246
	50	0.265	0.235	0.240	0.237	0.226
	100	0.283	0.198	0.248	0.239	0.239
	200	0.242	0.220	0.243	0.232	0.246
	300	0.298	0.204	0.231	0.254	0.217
	400	0.238	0.201	0.247	0.237	0.232
	*p*-value	0.3852	0.5411	0.9604	0.9792	0.8188

* Different lowercase letters are significantly different among treatment at *p* ≤ 0.05 level.

## Data Availability

The original contributions presented in the study are included in the article/Supplementary Materials, further inquiries can be directed to the corresponding author.

## Supplementary Materials

The following supporting information can be downloaded at: https://www.mdpi.com/article/10.3390/metabo14090470/s1, Figure S1: Deconvoluted peaks of Brussels sprouts’s shoot between wavenumber 1100 and 1900 cm^−1^ after 8 days of NaCl treatment.; Figure S2: Deconvoluted peaks of Brussels sprouts’s shoot between wavenumber 1100 and 1900 cm^−1^ after 10 days of NaCl treatment.
