# Supplementary material for: Application of ATR-FTIR Spectroscopy for Analysis of Salt Stress in Brussels Sprouts
Source: Metabolites. 2024 Aug 26;14(9):470. doi: 10.3390/metabo14090470 (PMC11433683; doi:10.3390/metabo14090470)

Figure S1. Deconvoluted peaks of brussels sprouts's shoot between wavenumber 1100 and 1900 $cm^{-1}$ after 8 days of NaCl treatment (black line, original FTIR spectrum; red line, cumulative spectrum of deconvoluted peaks; blue line, deconvoluted peak; 1630 $cm^{-1}$, amide Ⅰ; 1540 $cm^{-1}$, amide Ⅱ; 1250 $cm^{-1}$, amide Ⅲ).

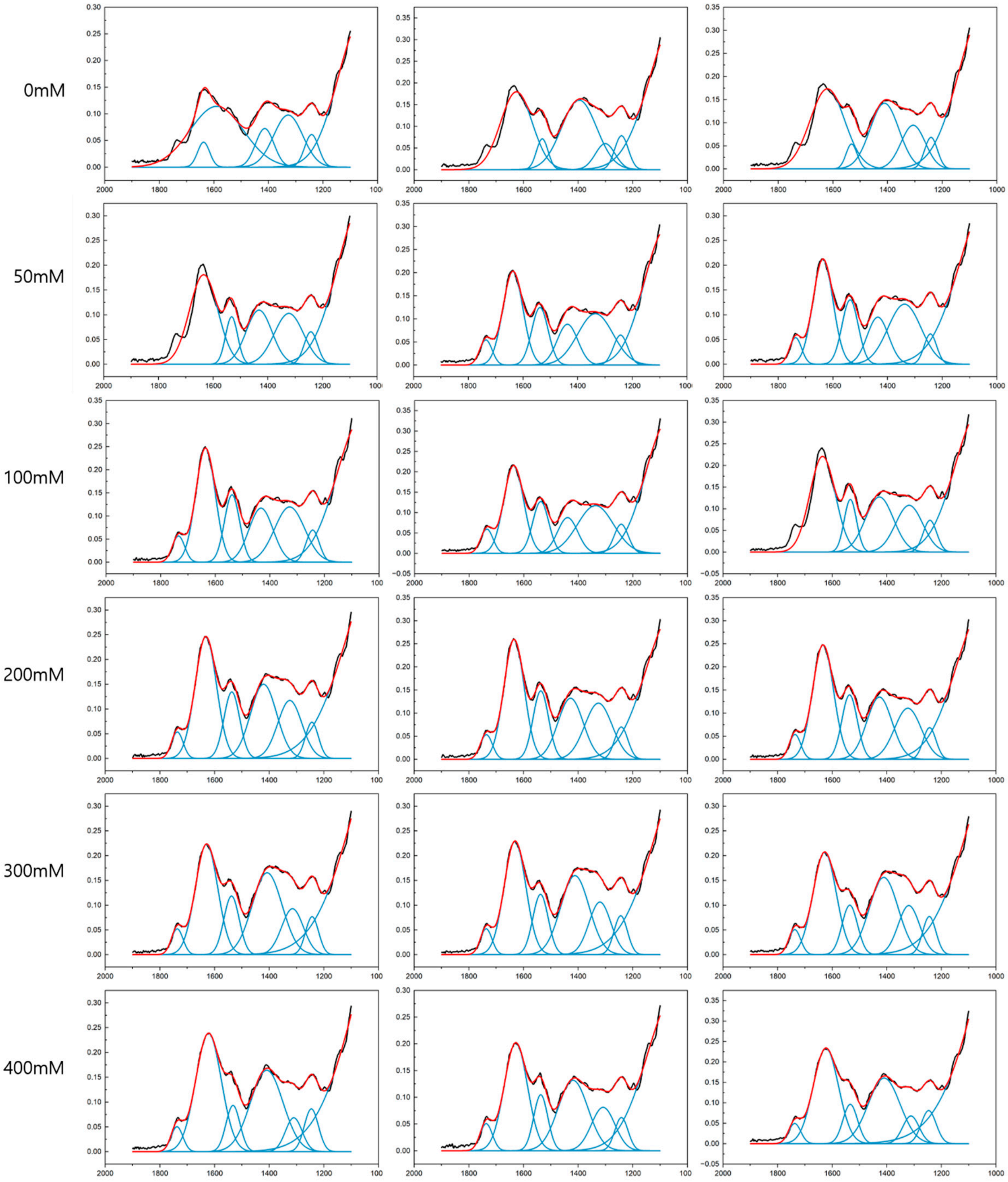

Figure S2. Deconvoluted peaks of brussels sprouts's shoot between wavenumber 1100 and 1900 cm$^{-1}$ after 10 days of NaCl treatment (black line, original FTIR spectrum; red line, cumulative spectrum of deconvoluted peaks; blue line, deconvoluted peak; 1630 cm$^{-1}$, amide Ⅰ; 1540 cm$^{-1}$, amide Ⅱ; 1250 cm$^{-1}$, amide Ⅲ).

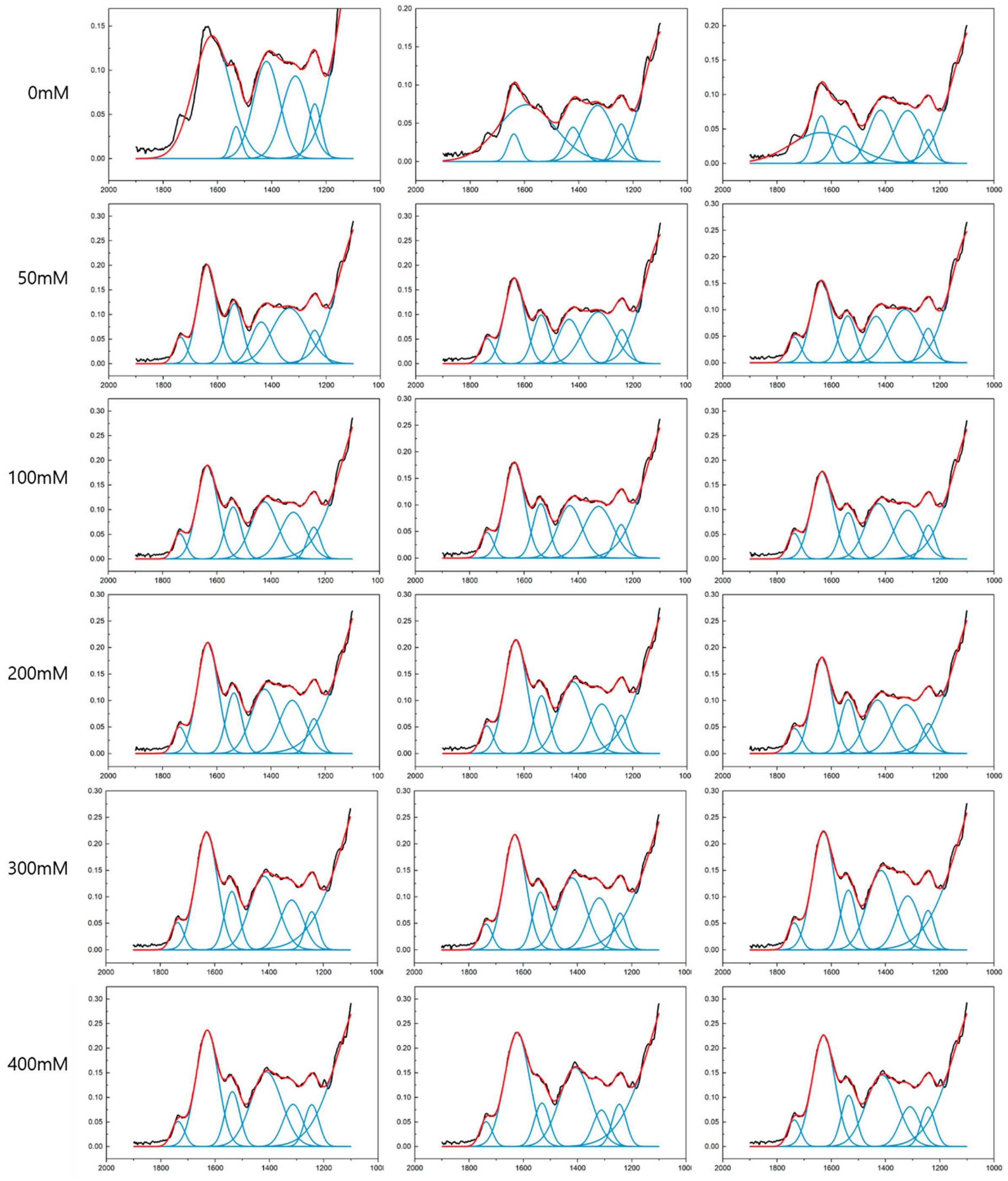

Supplement: Supplementary file 1 [file metabolites-14-00470-s001.zip › metabolites-3159851-supplementary.pdf]
